# *Escherichia marmotae* Dominates the Global Distribution of *cdtABC* Carriers in the Human Gut

**DOI:** 10.3390/microorganisms14102244

**Published:** 2026-10-03

**Authors:** Zhuo Pan, Ziang Wang, Songlin Zhao, Guangjun Chen, Daoyuan Wu, Qingxin Xia

**Affiliations:** Department of Pathology, The Affiliated Cancer Hospital of Zhengzhou University & Henan Cancer Hospital, Zhengzhou 450008, China; panzhuoddv@163.com (Z.P.);

**Keywords:** cytolethal distending toxin, bacterial genotoxin, gut microbiome, *Escherichia marmotae*, colorectal cancer

## Abstract

The gut microbiota can influence colorectal cancer through microbial metabolism, immune regulation and direct damage to host DNA. Previous experimental studies have shown that cytolethal distending toxin (CDT) can damage host DNA and promote colorectal tumorigenesis in model systems. However, the distribution of bacteria carrying intact *cdtABC* systems in human gut populations remains poorly characterized. Here, we integrated systematic screening of 60,008 complete bacterial genomes with analyses of 12,238 human gut metagenomes from 45 countries. We identified 871 intact *cdtABC* loci in 783 genomes, revealing a taxonomically restricted but genetically diverse carrier landscape. Carriers were detected worldwide, but the population signal was strongly dominated by an *Escherichia marmotae* lineage, a pattern that remained robust across minimum abundance thresholds. Most carrier-detected samples contained a single carrier, indicating pronounced lineage dominance. After carrier species were removed from the taxonomic profiles, the surrounding microbiome consistently distinguished samples with and without carriers across studies. This community signal was strongest for *E. marmotae* but remained detectable for other carriers and involved reproducible associations with *Escherichia*, *Faecalibacterium* and other gut taxa. By connecting intact loci with carrier lineages, global gut distribution and microbial community context, our study extends CDT research beyond isolated bacterial strains. These findings provide a basis for prioritising *cdtABC* carriers for functional validation and evaluating their potential relevance to colorectal cancer.

## 1. Introduction

The gut microbiota is increasingly recognized as a functional component of human physiology, contributing to epithelial homeostasis, immune regulation and host metabolism. Colorectal cancer (CRC) represents one of the clearest disease contexts in which host–gut microbiota interactions may influence pathogenesis [1]. Large-scale metagenomic analyses have identified reproducible microbial signatures associated with CRC across independent cohorts and geographical populations, with recent studies further resolving reproducible signatures at the strain level [2,3,4,5]. Mechanistic studies have shown that selected gut bacteria can shape immune environments that favour tumour development, form carcinogenic biofilms or directly damage host DNA [6,7,8]. Among the bacterial genotoxins implicated in CRC, cytolethal distending toxin (CDT) is of particular interest because experimental studies have demonstrated that *Campylobacter jejuni* promotes colorectal tumorigenesis in a CDT-dependent manner and that *C. jejuni*-derived CDT can enhance CRC metastasis [9,10].

CDT is a tripartite AB_2_-type genotoxin encoded by the adjacent *cdtA*, *cdtB* and *cdtC* genes in diverse Gram-negative bacteria [11]. CdtA and CdtC constitute the receptor-binding and delivery module, whereas CdtB is the catalytically active subunit of the toxin [12,13]. Following binding of the holotoxin to the cell surface and endocytic uptake, CdtB undergoes retrograde intracellular trafficking and ultimately reaches the nucleus [14,15]. Its nuclease activity induces DNA lesions that can be converted into double-strand breaks during S phase, triggering sustained DNA damage responses, cell-cycle arrest, senescence or cell death [16,17]. Cells that survive sublethal CDT exposure may retain and transmit genomic instability to their progeny, as observed in human colorectal cell lines and organoids [18,19]. However, current research is focused on a limited number of bacterial isolates, toxin variants and cellular models. It therefore remains unclear which bacterial lineages harbour intact *cdtABC* systems and how *cdtABC*-carrier strains are distributed across human gut populations.

In this study, we established an integrated framework linking bacterial genome mining with analyses of human gut metagenomes at the population level. We first screened 60,008 complete RefSeq bacterial genomes to identify strictly defined *cdtABC* loci. We then examined 4612 genomes from the Unified Human Gastrointestinal Genome reference collection and quantified 17 predicted carriers across 12,238 gut metagenomes from 65 studies in 45 countries. Finally, we removed the carrier taxa from the community profiles and examined their associations with other gut microorganisms using machine learning, SHAP interpretation and FastSpar analysis. Our analyses revealed a genetically diverse *cdtABC* landscape, a geographically widespread gut signal dominated by an *E. marmotae* carrier, and reproducible associations with the surrounding microbial community. This work maps predicted *cdtABC* carriers and their distribution in the human gut. It provides a basis for future studies of CDT in human health.

## 2. Materials and Methods

### 2.1. Identification of Candidate Cdt Genes and Adjacent Loci

Complete bacterial genome assemblies were obtained from the NCBI RefSeq database [20] on 11 February 2026. We retained assemblies annotated by NCBI RefSeq and excluded metagenome-assembled genomes, atypical assemblies and genomes from large multi-isolate projects. Genome sequences, protein sequences, GFF3 annotations and sequence reports were downloaded using NCBI Datasets, yielding an initial catalogue of 60,008 assemblies. Protein FASTA files were checked for gzip integrity before analysis. Protein sequences from these genomes were pooled, and exact amino acid duplicates were collapsed using MMseqs2 at 100% sequence identity and 100% bidirectional coverage [21]. A sequence-to-occurrence table was retained to map every non-redundant protein back to its original protein identifier and genome accession.

Non-redundant proteins were annotated against the KOfam profile hidden Markov model database using KofamScan [22]. Only assignments exceeding the KO-specific adaptive score threshold were retained. K11013, K11014 and K11015 were used as the initial signatures of CdtA, CdtB and CdtC, respectively. These assignments were expanded to their original protein occurrences and linked to CDS coordinates via RefSeq GFF3 files, followed by Conserved Domain Database (CDD) validation requiring an *E*-value ≤ 1 × 10^−5^ and model coverage ≥ 70%.

An adjacent *cdtABC* locus was defined as three consecutive CDSs located on the same contig and strand, containing one copy each of K11013, K11014 and K11015. The accepted genomic order was K11013–K11014–K11015, corresponding to the transcriptional order *cdtA*–*cdtB*–*cdtC*. Adjacency was determined from consecutive CDS indices, with intergenic distance ≤ 500 bp.

### 2.2. Conserved Domain Analysis

Candidate Cdt proteins were searched against the NCBI Conserved Domain Database using RPS-BLAST [23]. The subunit-specific models cd23414, cd09081 and cd23413 were required for CdtA, CdtB and CdtC, respectively. An expected domain was considered complete when its E-value was ≤1 × 10^−5^ and its alignment covered at least 70% of the corresponding CDD model. InterProScan was additionally used to obtain independent protein-family and domain annotations [24]. InterPro annotations were used as supporting evidence but did not replace the subunit-specific CDD criteria.

Proteins containing internal stop codons or more than 5% ambiguous residues were flagged as sequence-quality failures. An adjacent locus was classified as a complete strict locus when all three proteins contained their expected complete CDD domains and passed sequence-quality assessment. Loci satisfying the domain criteria but containing an extreme protein-length value were retained as review-length variants and flagged in the catalogue. Loci with absent, weak or partial expected-domain matches were excluded from the final carrier set. A carrier genome was defined as a genome containing at least one complete strict locus.

### 2.3. Cdt Gene Co-Occurrence and Taxonomic Distribution

For the genome-level co-occurrence analysis, all proteins assigned to K11013, K11014 or K11015 were evaluated independently using the same CDD E-value and model-coverage criteria. A subunit was considered present in a genome when at least one protein contained the corresponding complete CDD model. The seven possible CdtA, CdtB and CdtC presence combinations were enumerated from the resulting binary genome-by-subunit matrix. Locus copy number was defined as the number of complete strict loci detected in each genome. Genus-level analyses reported both carrier prevalence and the number of non-redundant *cdtABC* operon haplotypes.

### 2.4. Phylogenomic Analysis and Operon Dereplication

A genome-scale phylogeny was inferred for genomes containing at least one preliminary K11013, K11014 or K11015 assignment using PhyloPhlAn 3.0 with the high-diversity and fast settings [25]. The resulting phylogeny was annotated with taxonomic assignments, CdtA, CdtB and CdtC domain-supported presence, adjacent-locus presence, complete-strict-locus presence, total KO-hit number and locus copy number. Tree visualization and annotation were performed using Interactive Tree of Life v6 [26].

For locus-level dereplication, terminal stop symbols were removed and the CdtA, CdtB and CdtC amino acid sequences from each complete strict locus were normalized to A–B–C order. Exact operon haplotypes were defined by the SHA-256 digest of the three sequences joined in A–B–C order. Two loci were assigned to the same haplotype only when all three subunit sequences were identical. These non-redundant operon haplotypes were used for genus-level diversity summaries and subsequent CdtABC sequence phylogenetic analyses.

### 2.5. Operon Haplotype Definition and Phylogenetic Analysis

The 871 complete *cdtABC* loci were dereplicated using the full-length amino acid sequences of all three subunits. CdtA, CdtB and CdtC sequences were aligned separately using MAFFT v7.526 with the automatic strategy [27], and alignments were trimmed using the automated procedure in trimAl. The three trimmed alignments were concatenated while retaining CdtA, CdtB and CdtC as separate partitions. Maximum-likelihood trees were inferred for each subunit and for the partitioned concatenated alignment using IQ-TREE v3.1.3 [28]. Substitution models were selected using ModelFinder, with partition merging permitted for the concatenated analysis [29]. Branch support was assessed using 1000 ultrafast bootstrap and 1000 SH-like approximate likelihood-ratio test replicates. The trees were treated as unrooted, and taxonomic assignments and haplotype occurrence counts were mapped to the terminal nodes. Exact haplotypes detected in genomes assigned to more than one species were identified from the locus-to-haplotype mapping table.

### 2.6. Neighbourhood and Mobile Element Annotation Analysis

We analysed ten CDS positions on each side of every *cdtABC* locus to obtain a standardized view of the immediate protein-coding neighbourhood across genomes with different gene lengths and architectures. This window supports comparison of recurrent local features. For each complete *cdtABC* locus, coding sequences were extracted from the corresponding RefSeq GFF file for the ten positions upstream and downstream of the operon. Relative positions were reoriented according to the transcriptional direction of *cdtABC*, such that negative and positive values represented upstream and downstream coding sequences, respectively. Neighbouring proteins were assigned KEGG Orthologs using KofamScan and KO-specific adaptive score thresholds [22]. Where a KO assignment was available, it was used to define the neighbouring gene family; otherwise, the normalized RefSeq product annotation was retained. For the position-resolved summary, recurrent non-hypothetical families were ranked by the number of distinct *cdtABC* loci in which they occurred, and the 16 highest-ranking families were displayed. Mobile element functions were annotated by searching the flanking protein sequences against MobileOG-db beatrix 1.6 using blastp. Hits were retained when they had an E-value ≤ 1 × 10^−5^, amino acid identity ≥ 30% and query coverage ≥ 70%. Functional categories were assigned according to the corresponding MobileOG annotations.

### 2.7. Identification of cdtABC Carrier Genomes in UHGG

Protein-coding sequences from the 4612 Unified Human Gastrointestinal Genome (UHGG) representative genomes included in the abundance resource were annotated using KofamScan as mentioned before. Taxonomic assignments were obtained from the GTDB lineage annotations accompanying the UHGG resource.

### 2.8. Metagenomic Cohort Integration and Carrier Quantification

Genome abundance profiles, total read counts and sample metadata were obtained for 12,238 human gut metagenomes from 65 studies and 45 countries, as compiled previously [30]. While standardized bioinformatic profiling against unified UHGG species representatives mitigates analytical discrepancies, the metagenomes originated from independent public studies with heterogeneous sample collection, storage, DNA extraction and sequencing protocols. We therefore preserved study boundaries through leave-one-study-out validation or within-study estimation. Samples were matched across the abundance matrix, metadata and sequencing depth tables using exact sample identifiers. Rows corresponding to the 17 independently identified carrier genomes were extracted from the UHGG abundance matrix. Because carrier status had been established from the representative genomes, a sample was considered to contain a given carrier at baseline when at least one read was assigned to the corresponding UHGG. To assess sensitivity to low-abundance read assignments, carrier detection was recalculated using minimum carrier-specific abundance thresholds of 10, 25, 50, 100, 250 and 500 CPM. At each threshold, we recalculated the detection prevalence of any carrier, the *E. marmotae* representative and other carriers. We also recalculated the proportion of carrier-detected samples containing a single carrier, the proportion dominated by the *E. marmotae* representative and its contribution to retained carrier reads. Country prevalence estimates at 100 CPM were compared with the baseline estimates using Spearman correlation. Because five candidate carrier genomes had zero assigned reads across all samples, abundance, richness and dominance calculations were performed over the 12 detected carrier genomes. The total carrier abundance in a sample was calculated as follows:

$$\mathrm{Carrier}\;{\mathrm{CPM}}_{j}=\frac{\sum_{i=1}^{12}x_{\mathrm{ij}}}{T_{j}}\times 10^{6}$$

Carrier-specific abundance was calculated as follows:

$$\mathrm{CP}M_{\mathrm{ij}}=\frac{x_{\mathrm{ij}}}{T_{j}}\times 10^{6}$$

The contribution of each carrier to the total carrier signal was calculated by dividing its summed read count across all samples by the summed read count of all 17 carriers. The label *E. marmotae* refers to the UHGG representative genome GUT_GENOME140808 and its accompanying species-level taxonomic assignment. Because the abundant resource was derived from short-read metagenomes, assignments to this representative cannot exclude contributions from very closely related Escherichia lineages.

### 2.9. Geographic Distribution and Carrier Structure Within Samples

Carrier prevalence was summarized for the complete cohort and separately by study, country and continent. For each stratum, prevalence was calculated as the number of samples containing at least one carrier divided by the total number of samples in that stratum. Carrier-specific prevalence within each continent was calculated using the same definition. All 65 studies were retained in the overall prevalence summaries. Seven studies contained samples from more than one continent and were therefore excluded only from study-level summaries that required assigning each entire study to a single continent. Country boundaries used for geographic visualization were obtained from the Natural Earth 1:110 million cultural vector dataset. Country estimates describe the available metagenomic collection and vary substantially in precision because sample numbers differ among countries. They are not weighted population prevalence estimates, and values from countries represented by few samples should be interpreted cautiously.

Carrier richness in samples was defined as the number of detected carrier genomes:

$$R_{j}=\sum_{i=1}^{12}I\left(x_{\mathrm{ij}}>0\right)$$

For samples containing at least one carrier, the dominant carrier was the genome with the largest assigned read count, and its fractional contribution was calculated as follows:

$$D_{j}=\frac{\max_{1\leq i\leq 12}x_{\mathrm{ij}}}{\sum_{i=1}^{12}x_{\mathrm{ij}}}$$

Samples were further divided into four mutually exclusive states: no detected carrier, *E. marmotae* only, one or more other carriers without *E. marmotae*, and *E. marmotae* together with at least one other carrier. Richness and dominant carrier fraction were summarized only among samples containing at least one carrier. All analyses were descriptive and no formal comparisons of prevalence among countries or continents were performed.

Samples were stratified using the available health and disease metadata. Detection of any candidate *cdtABC* carrier was defined as at least one read assigned across the 17 carrier genomes, and abundance was calculated as their summed counts per million reads (CPM). For each category, we summarized detection percentages with Wilson 95% confidence intervals and median CPM with interquartile ranges among detected samples. CRC and healthy samples were compared within studies. Detection odds ratios were estimated with a continuity correction of 0.5 applied to all four cells when a zero cell was present; studies with no detections in either group were excluded. Abundance comparisons used the difference in mean log_10_(CPM + 1), retaining zeros, with sampling variance calculated as the sum of the group mean variances. These comparisons required at least three samples per group and a nonzero sampling variance. Study estimates were combined using DerSimonian–Laird random effects models with normal 95% confidence intervals. *p* values were two-sided and exploratory. Disease category summaries were interpreted descriptively because study representation differed between categories.

### 2.10. Construction of the Carrier-Depleted Community Matrix

Read counts for 4612 Unified Human Gastrointestinal Genome (UHGG) representative genomes across 12,238 human gut metagenomes were matched to sample metadata and total sequencing depth using exact sample identifiers. To prevent direct carrier leakage, the 17 *cdtABC* carrier genomes and all additional UHGGs assigned to the same normalized Genome Taxonomy Database species as these carriers were excluded. The remaining genomes were aggregated at the species level; genomes lacking species-level assignments were retained as genome-specific unclassified features. Taxa detected in at least 2% of samples and with non-zero total counts were retained, yielding 321 non-carrier taxon features. For machine learning and association analyses, read counts were converted to counts per million total reads (CPM) and transformed as log(1 + CPM).

### 2.11. Classification of cdtABC Carrier Status

Three carrier-status definitions were evaluated. In the primary analysis, samples containing reads assigned to any of the 17 carriers were classified as carrier-detected, whereas samples without carrier reads were classified as carrier-undetected. In the first sensitivity analysis, samples containing only the *E. marmotae* carrier were removed, whereas samples containing *E. marmotae* together with another carrier remained in the positive group. In the second sensitivity analysis, all *E. marmotae*-detected samples were excluded, and samples containing one or more other carriers were compared with carrier-undetected samples.

Carrier status was predicted from the carrier-depleted community matrix using an XGBoost classifier. Generalizability was evaluated by leave-one-study-out validation across 65 studies, with each study held out once for testing and the remaining studies used for model training. Within each training fold, taxa present in at least 1% of training samples were retained, and no more than 300 features were selected according to abundance variance. Models comprised 250 trees with a maximum depth of 3, a learning rate of 0.05, sample- and feature-subsampling rates of 0.8, and a binary logistic objective. Samples were weighted inversely by study size and balanced between positive and negative classes. All preprocessing, feature selection and model fitting were performed independently within each training fold.

Model performance was calculated from pooled held-out predictions using the area under the receiver operating characteristic curve (AUROC) and the area under the precision–recall curve (AUPRC). Confidence intervals were estimated using 2000 study-cluster bootstrap replicates, in which studies rather than individual samples were sampled with replacement. A fixed random seed of 20260811 was used for model fitting, held-out sample selection and bootstrap analyses.

### 2.12. Identification of Co-Colonizers and Co-Excluders

We identified *cdtABC*-associated taxa using complementary cross-study SHAP and FastSpar analyses. Feature contributions were first quantified using TreeExplainer and SHapley Additive exPlanations (SHAP). Models were fitted independently within each leave-one-study-out fold, and SHAP values were calculated exclusively for samples from the corresponding held-out study. Up to 150 samples were selected from each held-out study, with carrier-detected and carrier-undetected samples balanced whenever possible. For each species in each study, the SHAP direction was defined as the difference in mean SHAP value between samples in which the species was present and those in which it was absent. The overall SHAP direction was defined as the median of these study-specific differences, whereas feature importance was quantified as the mean absolute SHAP value weighted by the number of explained samples in each study. A species was considered SHAP-stable if its mean absolute SHAP importance was within the top 5%, its association direction was concordant in at least 70% of informative studies, and its direction could be estimated in at least 10 studies. The highest-ranked stable features in each direction were retained, together with prespecified *Faecalibacterium* taxa that satisfied the same stability criteria, yielding 17 SHAP-screened taxa. SHAP was used only for feature ranking and cross-study stability assessment and was not treated as a formal significance test.

FastSpar was used as a complementary compositional correlation analysis41. The input count table contained raw read counts for all 321 non-carrier taxa and a pseudo-feature representing the summed raw reads assigned to the 17 *cdtABC* carrier genomes in each sample. FastSpar was run with 50 inference iterations and 20 exclusion iterations. Statistical support was estimated using 999 bootstrap replicates, and the resulting *p* values were adjusted across all 321 non-carrier taxa using the Benjamini–Hochberg procedure. A taxon was retained as a final *cdtABC* associated candidate only when the FastSpar correlation was directionally concordant with its held-out SHAP effect and the FastSpar association had an FDR-adjusted q < 0.05. Thirteen taxa met these joint criteria, including eight positively associated and five negatively associated taxa, operationally termed co-colonizers and co-excluders, respectively. Throughout this analysis, co-colonizers and co-excluders refer only to taxa showing positive and negative statistical associations with *cdtABC* carrier detection, respectively. These operational labels describe directions of association and do not establish direct facilitation, competition, exclusion, or other ecological interactions. The observed patterns may also reflect shared associations with host, environmental, dietary, or study-specific factors.

### 2.13. Cross-Study Evaluation of cdtABC Carrier Associations

Associations were evaluated under the any-carrier, remove *E. marmotae*-only and other-carrier-only definitions. Candidate abundances were expressed as log(1 + CPM). Within each study, Spearman’s rank correlation was calculated between taxon abundance and binary carrier status. A study was included when it contained at least 20 samples, at least three carrier-detected and three carrier-undetected samples, and variation in the abundance of the evaluated taxon. The minimum group size was used to avoid estimating within-study correlations from extremely sparse strata. Consequently, associations involving rare carriers were informed only by studies with sufficient carrier detections.

Abundance-gradient associations were evaluated among carrier-detected samples by calculating the within-study Spearman correlation between candidate abundance and log(1 + total carrier CPM). Studies were required to contain at least eight carrier- detected samples and variation in both candidate and total carrier abundance. Study-specific Spearman coefficients were transformed using Fisher’s transformation. Effects were combined using a random-effects model, with between-study variance estimated using the DerSimonian–Laird procedure.

At least three eligible studies were required to estimate a pooled association. For the primary any-carrier analysis, *p* values were adjusted across all 321 non-carrier taxa using the Benjamini–Hochberg procedure. A taxon was classified as globally robust when it satisfied the SHAP stability criteria, had q < 0.05 in both the globally corrected FastSpar and pooled Spearman analyses, and showed concordant directions across SHAP, FastSpar and pooled Spearman estimates. Ten taxa met these criteria. These taxa were subsequently evaluated under the remove *E. marmotae*-only and other-carrier-only definitions, with *p* values adjusted across the ten taxa separately within each sensitivity analysis. An FDR-adjusted q < 0.05 was considered statistically significant.

To evaluate the loss of statistical sensitivity resulting from the smaller carrier-detected groups, the carrier-detected samples from the complete analysis were randomly downsampled without replacement, while all carrier-undetected samples were retained. For each of the ten globally robust taxa, the study-specific associations were recalculated and pooled using the same random effects procedure in 250 repetitions. Empirical recovery probability was defined as the proportion of repetitions in which the pooled association had nominal *p* < 0.05 and the same direction as the complete analysis.

## 3. Results

### 3.1. Genome Scale Screening Expands the Catalogue of Predicted cdtABC Carriers

**Figure 1:**
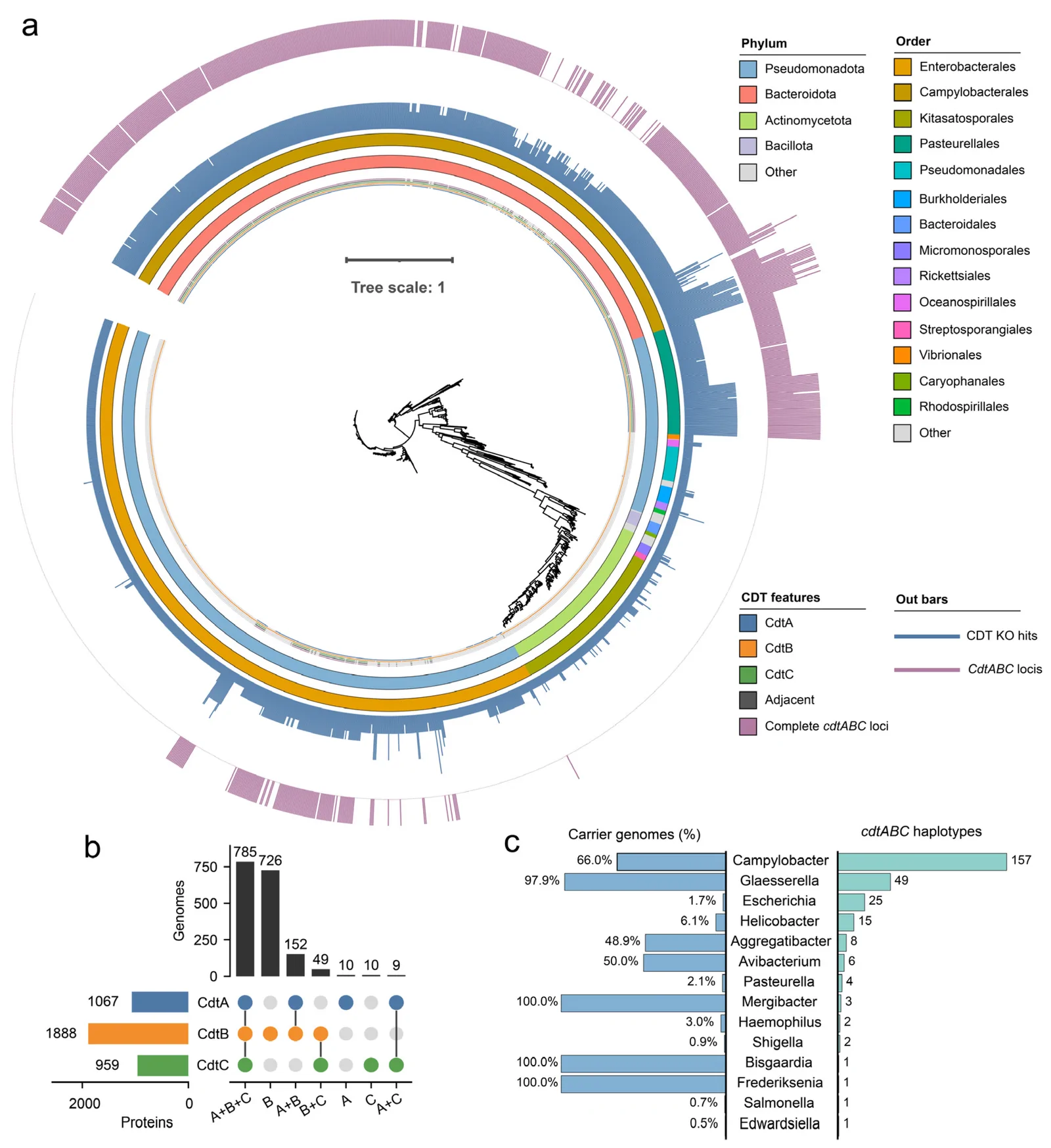
Genomic distribution and taxonomic landscape of *cdtABC* carriers. (**a**) Circular phylogeny of 1755 complete RefSeq bacterial genomes containing at least one KO-assigned CdtA, CdtB or CdtC signal. The tree was reconstructed from conserved genome-wide marker proteins. Each terminal branch represents one genome, and branch lengths indicate inferred phylogenetic distance in substitutions per site, according to the scale bar. From the centre outward, the five binary tracks indicate the presence of a CdtA KO hit, a CdtB KO hit, a CdtC KO hit, an adjacent *cdtABC* candidate, and a complete *cdtABC* locus, respectively. Filled symbols indicate the presence of the corresponding feature in a genome. The subsequent colour strips denote phylum and order assignments. The two outer bar tracks show the total number of CdtA, CdtB and CdtC KO hits and the number of complete *cdtABC* loci detected in each genome, respectively. (**b**) UpSet plot summarizing co-occurrence patterns of CdtA, CdtB and CdtC. Vertical bars show the number of genomes belonging to each subunit combination, and horizontal bars show the total number of detected proteins for each CDT subunit. (**c**) Genus-level distribution of complete *cdtABC* carriers. Left-facing bars show the percentage of genomes within each genus classified as *cdtABC* carriers, whereas right-facing bars show the number of exact sequence-dereplicated cdtABC haplotypes detected in that genus. Genera are ordered by decreasing cdtABC haplotype count.

To establish a genome catalogue of potential *cdtABC* carriers, we screened 60,008 complete RefSeq bacterial genomes using the KOfam hidden Markov model profiles (K11013, K11014 and K11015), retaining only hits exceeding the KO-specific adaptive score threshold. Candidates were then subjected to Conserved Domain Database (CDD) validation (*E*-value ≤ 1 × 10^−5^, coverage ≥ 70%) and genomic context filtering (intergenic distance ≤ 500 bp). The initial screen identified 3937 proteins assigned to CdtA, CdtB or CdtC in 1755 genomes spanning seven phyla and 28 orders (Figure 1a, Table S1). CDD analysis supported 1067 CdtA, 1888 CdtB and 959 CdtC proteins. At the genome level, 785 genomes encoded all three domain subunits, whereas 726 encoded CdtB alone, demonstrating the need to distinguish intact operons from isolated subunit homologues (Figure 1b). We therefore required CdtA, CdtB and CdtC to occur as consecutive coding sequences on the same contig and strand, in *cdtABC* genomic order. This positional filter identified 872 adjacent loci in 784 genomes. Exclusion of one locus containing a partial CdtB domain resulted in a final catalog of 871 complete *cdtABC* loci (Table S2) distributed across 783 predicted carrier genomes (Table S3; Supplementary Figure S1).

The 783 carrier genomes represented 63 species but were concentrated in 14 genera belonging to three orders (Figure 1c). Exact dereplication of the concatenated CdtA, CdtB and CdtC amino acid sequences reduced the 871 loci to 275 distinct operon haplotypes. *Campylobacter* formed the largest carrier group, with *cdtABC* detected in 544 of 824 genomes (66.0%), comprising 588 loci and 157 of the 275 haplotypes. *Glaesserella* combined near-complete genus-level prevalence with extensive locus duplication: 46 of 47 genomes (97.9%) were carriers, containing 90 loci and 49 haplotypes. 44 of these carrier genomes contained multiple loci, corresponding to an average of 1.96 loci per carrier genome. In contrast, *Escherichia* showed a low carrier prevalence of 1.71% but still contributed 91 carrier genomes and 25 haplotypes, indicating appreciable sequence diversity within a sparsely distributed trait. Carrier prevalence was also high in *Aggregatibacter* (48.9%) and *Avibacterium* (50.0%). This genome-scale catalog extends the sequence and taxonomic space of experimentally characterized CDT systems to 783 predicted carriers and 275 nonredundant operon sequences. These assignments indicate genetic potential and do not by themselves establish CDT production or activity.

### 3.2. Phylogenetic Lineage and Genomic Context Diversity of cdtABC Loci

**Figure 2:**
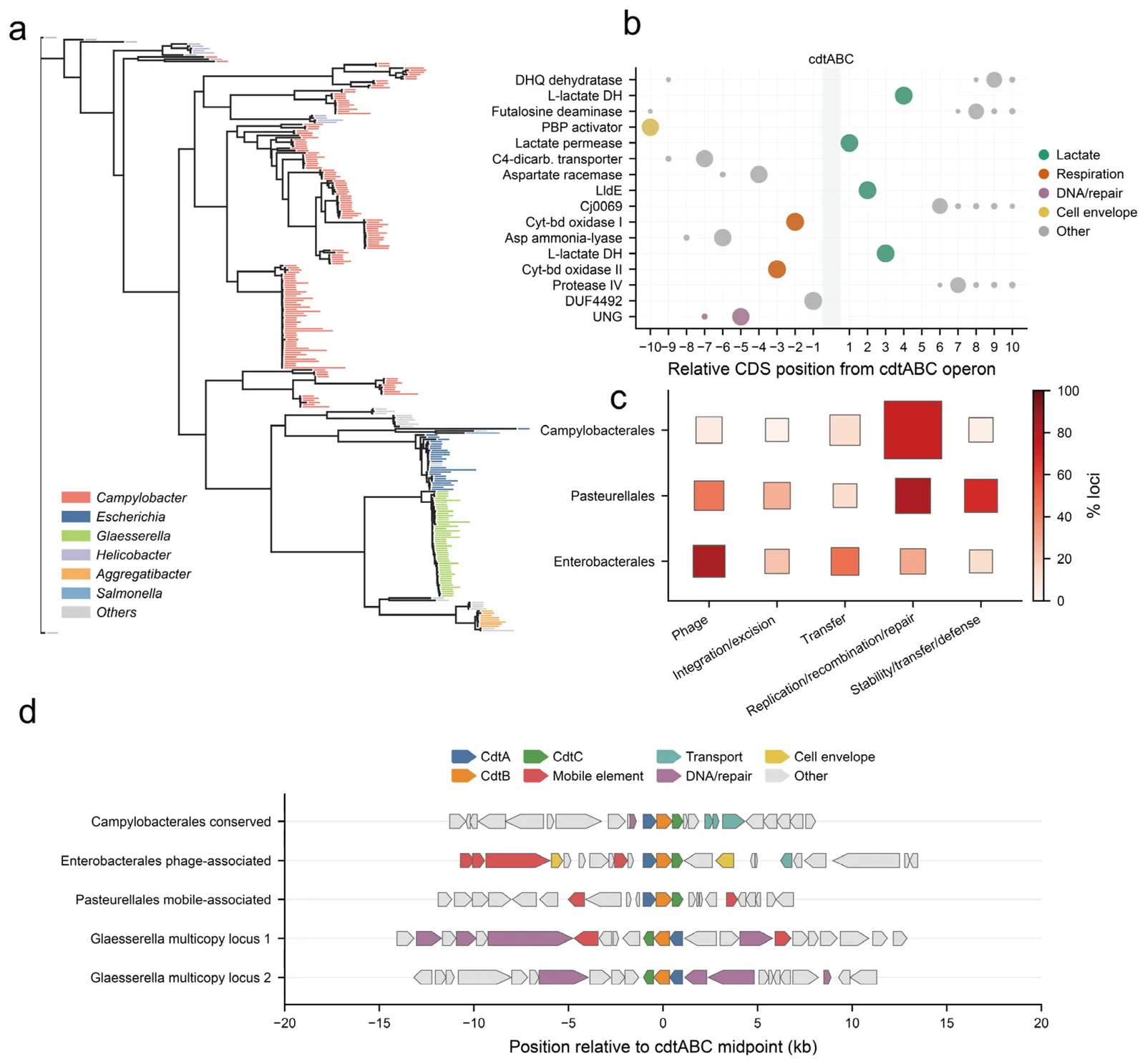
Evolutionary diversity and genomic contexts of *cdtABC* loci. (**a**) The maximum-likelihood tree is displayed as unrooted and is used to compare sequence divergence and taxonomic clustering. It does not imply the ancestral state or direction of *cdtABC* evolution. Terminal horizontal bars are colored according to the predominant carrier genus, and bar length reflects the number of loci represented by each haplotype. (**b**) Positional distribution of functional categories flanking *cdtABC* loci. Each circle represents a gene-family/relative-position combination, and color denotes its broad functional category. The x-axis indicates CDS position relative to the *cdtABC* operon after orientation according to the direction of operon transcription. Positions −10 to −1 are upstream and positions +1 to +10 are downstream. (**c**) Order-level distribution of MobileOG functional annotations in *cdtABC* neighbourhoods. The rectangle area represents the number of loci containing MobileOG hits in each functional category. (**d**) Representative genomic architectures of *cdtABC* loci.

Having established the taxonomic distribution of complete *cdtABC* loci, we next examined the phylogenetic relationships among their operon haplotypes and their local genomic contexts. The maximum likelihood phylogeny constructed from concatenated CdtA, CdtB and CdtC amino acid sequences showed broad genus-level clustering (Figure 2a). Pairwise comparisons showed strong correlations among the patristic-distance structures of the individual subunit and concatenated trees (Supplementary Figure S2). However, eight of the 275 exact operon haplotypes occurred in genomes assigned to more than one RefSeq species label, indicating that identical concatenated CdtABC amino acid sequences were not always confined to a single species label. In all eight cases, sharing remained within a single genus. Three involved a named species and genomes annotated only at the genus level, whereas the remaining five involved closely related Escherichia species.

We then characterized the ten coding sequences upstream and downstream of each complete *cdtABC* locus. Several gene families recurred at specific relative positions, including genes annotated to lactate metabolism, respiration, DNA processing and repair, and cell-envelope functions (Figure 2b). These patterns describe repeated local genomic associations but do not establish functional interactions between the neighbouring genes and the CDT system. MobileOG annotations of the flanking proteins also differed among the three carrier orders (Figure 2c). Enterobacterales loci showed the strongest phage and transfer signals, whereas Pasteurellales loci contained a broader distribution of MobileOG functional categories. Campylobacterales loci generally contained fewer phage, integration or excision, and transfer annotations within the analysed neighbourhoods.

Representative locus maps illustrated the genomic architectures underlying these order-level patterns (Figure 2d). These included a representative Campylobacterales architecture, an Enterobacterales locus with phage-related MobileOG annotations and a Pasteurellales locus containing several MobileOG-annotated proteins. Two loci from a multicopy Glaesserella genome also displayed distinct local organizations. Together, these analyses show that *cdtABC* diversity is organized at two complementary levels: operon sequences broadly track carrier taxonomy, whereas locus neighbourhoods differ in gene content and mobile-element-associated features.

### 3.3. Escherichia Marmotae Dominates the Globally Distributed cdtABC Carriers in the Human Gut

**Figure 3:**
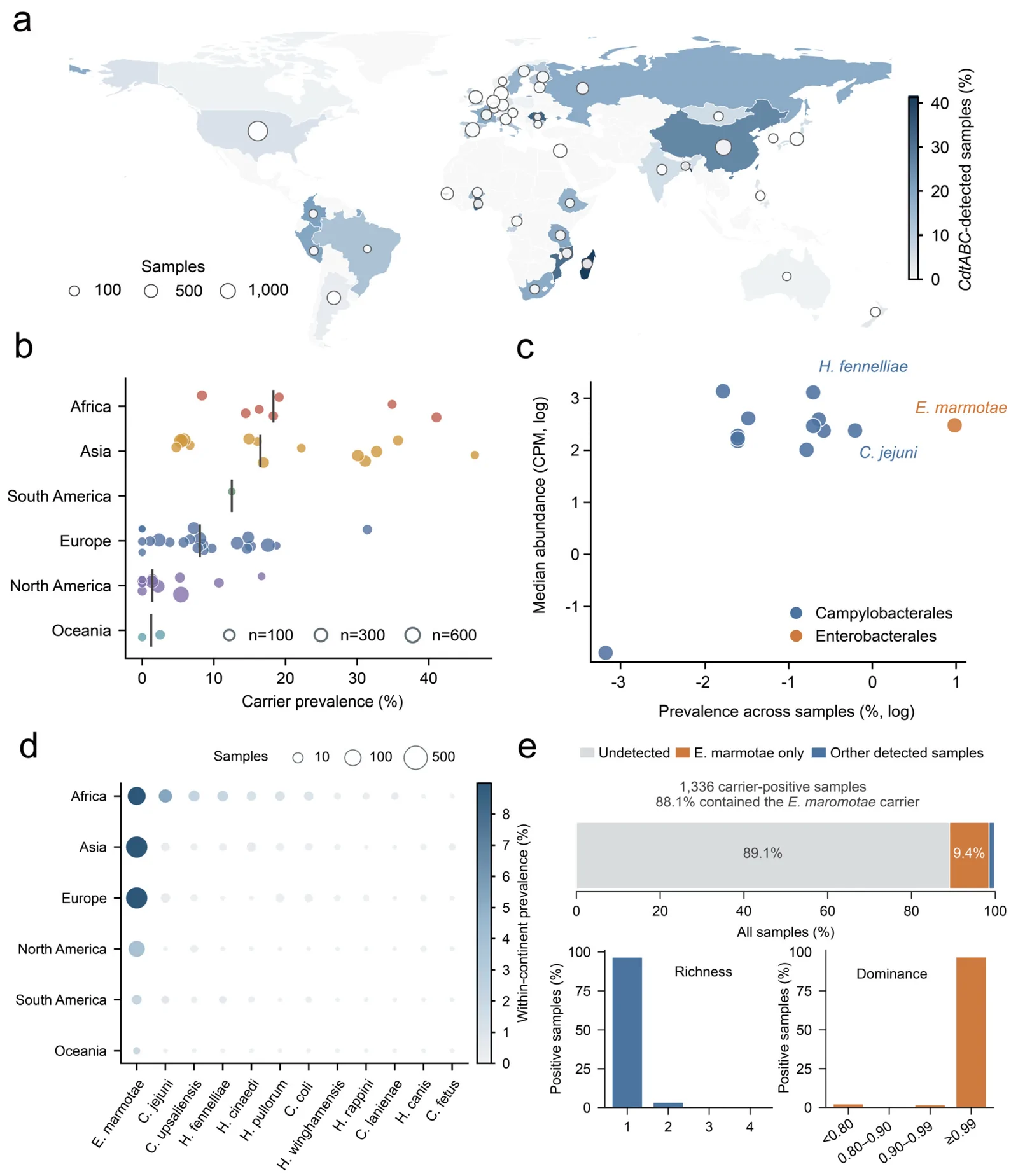
Global distribution and within-sample organization of *cdtABC* carriers in the human gut. (**a**) Global distribution of human gut metagenomes and *cdtABC* carrier prevalence. Circle area corresponds to the total number of metagenomic samples analysed per country, while color shading indicates the proportion of carrier-detected samples (prevalence, %). (**b**) Variation in carrier prevalence, grouped by continent. Each point represents one study, and point area represents study sample size. (**c**) Prevalence–abundance landscape of candidate *cdtABC* carrier genomes. Each point represents one candidate carrier genome. Its position indicates detection prevalence across all metagenomic samples on the x-axis and median abundance among samples in which that genome was detected on the y-axis; colour denotes bacterial order. (**d**) Distribution of the *cdtABC* carriers detected in the abundance matrix, ordered by decreasing continent prevalence. Dot area represents the number of carrier-detected samples, whereas color intensity represents carrier prevalence within each continent. (**e**) Composition and within-sample organization of carrier-detected samples. The upper stacked bar partitions all samples into carrier-undetected samples, samples containing only the *Escherichia marmotae* carrier, and the remaining carrier-detected samples. The lower-left plot shows carrier richness. The lower-right plot shows the dominant-carrier fraction. The world base map was generated using public domain geographic data from Natural Earth (https://www.naturalearthdata.com/).

To characterize the distribution of *cdtABC* carriers in the human gut, we independently screened 4612 Unified Human Gastrointestinal Genome (UHGG) representative genomes and identified 23 complete *cdtABC* loci in 17 carrier genomes (Table S4). We then quantified these carriers across 12,238 human gut metagenomes from 65 studies and 45 countries. Overall, carriers were detected in 1336 samples (10.92%), spanning 38 of the 45 surveyed countries and all six represented continents (Figure 3a, Supplementary Figure S3a). Carrier prevalence nevertheless varied markedly among countries and geographic regions, indicating that *cdtABC* carriers were globally distributed but unevenly represented across the surveyed gut. Moreover, stratification by health and disease status showed variation in carrier detection and abundance across the available sample categories (Supplementary Figure S4a,b). Carriers were detected in 13.42% of CRC samples and 11.69% of healthy samples. However, comparisons within six informative studies did not show a significant pooled difference in detection prevalence or abundance (Supplementary Figure S4c,d).

Carrier prevalence also varied substantially among studies, although carrier-detected samples were not confined to a small number of datasets. At least one *cdtABC* carrier-detected sample was detected in 56 of the 65 studies, with study-level prevalence ranging from 0 to 46.43% (Figure 3b, Supplementary Figure S3b). This cross-study distribution masked a pronounced imbalance among individual carriers. Only 12 of the 17 UHGG carriers were detected in the abundance matrix, and their prevalence spanned more than two orders of magnitude (Figure 3c, Table S4). The *E. marmotae* carrier was detected in 1177 samples (9.62%), compared with 76 samples for *Campylobacter jejuni* (0.62%) and 32 for *Campylobacter upsaliensis* (0.26%). It also accounted for 89.36% of the summed carrier reads and reached a median abundance of 302.16 counts per million total reads (CPM) when present. Conversely, several low-prevalence carriers reached high abundances in the small number of samples in which they were detected. For example, *Helicobacter cinaedi* was detected in 24 samples with a median abundance of 1292.31 CPM, whereas *Campylobacter fetus* was detected in only two samples with a median abundance of 1361.06 CPM. Thus, carriers that were rarely detected could still be abundant in individual carrier-detected samples, although the overall carrier signal across the cohort was dominated by *E. marmotae*.

The predominance of the *E. marmotae* carrier was not restricted to a single geographic region. It was detected across all six continents, with prevalence ranging from 20.02% in Africa, 14.66% in Asia and 9.52% in Europe to 3.92% in North America, 2.11% in South America and 1.92% in Oceania (Figure 3d). In contrast, most other carriers showed more restricted continental distributions: Africa and Asia each contained 10 of the 12 detected carriers, compared with eight in Europe, six in South America, three in North America and only one in Oceania. The second most prevalent carrier, *C. jejuni*, was detected predominantly in Africa, which accounted for 55 of its 76 carrier-detected samples. These continental distribution patterns showed that the prominence of *E. marmotae* was not attributable solely to a single regional cohort, whereas the remaining carriers were generally rarer and more geographically restricted.

*cdtABC*-detected samples were also characterized by low carrier richness and strong dominance. Of the 12,238 samples, 10,902 (89.08%) were carrier-undetected, 1155 (9.44%) contained only the *E. marmotae* carrier, 159 (1.30%) contained other carriers without *E. marmotae*, and 22 (0.18%) contained *E. marmotae* together with at least one additional carrier (Figure 3e). Accordingly, 1177 of the 1336 carrier-detected samples (88.10%) contained the *E. marmotae* carrier. Carrier richness was one in 1287 carrier-detected samples (96.33%), leaving only 49 samples with two or more detected carriers. Consistently, the most abundant carrier contributed at least 99% of the summed carrier abundance in 1288 carrier-detected samples (96.41%), resulting in median carrier richness and dominant-carrier fraction values of 1 and 1.00, respectively. To assess the robustness of these results, we repeated the analysis using minimum abundance thresholds of 10–500 CPM. Although overall carrier detection declined from 10.92% at baseline to 9.58% at 100 CPM and 4.00% at 500 CPM, the *E. marmotae* representative remained dominant, accounting for 84.46–88.48% of dominant carrier assignments and 89.35–89.41% of retained carrier reads across these thresholds. Country prevalence estimates at 100 CPM were also strongly correlated with the baseline estimates (Spearman’s ρ = 0.969; Supplementary Figure S5). Together, these findings define a highly skewed human gut *cdtABC* carrier landscape in which most carrier-detected samples contained a single dominant carrier and the population-wide pattern was largely driven by *E. marmotae*.

### 3.4. Cross-Study Community Modelling Identifies Microbial Correlates of cdtABC Carriers

**Figure 4:**
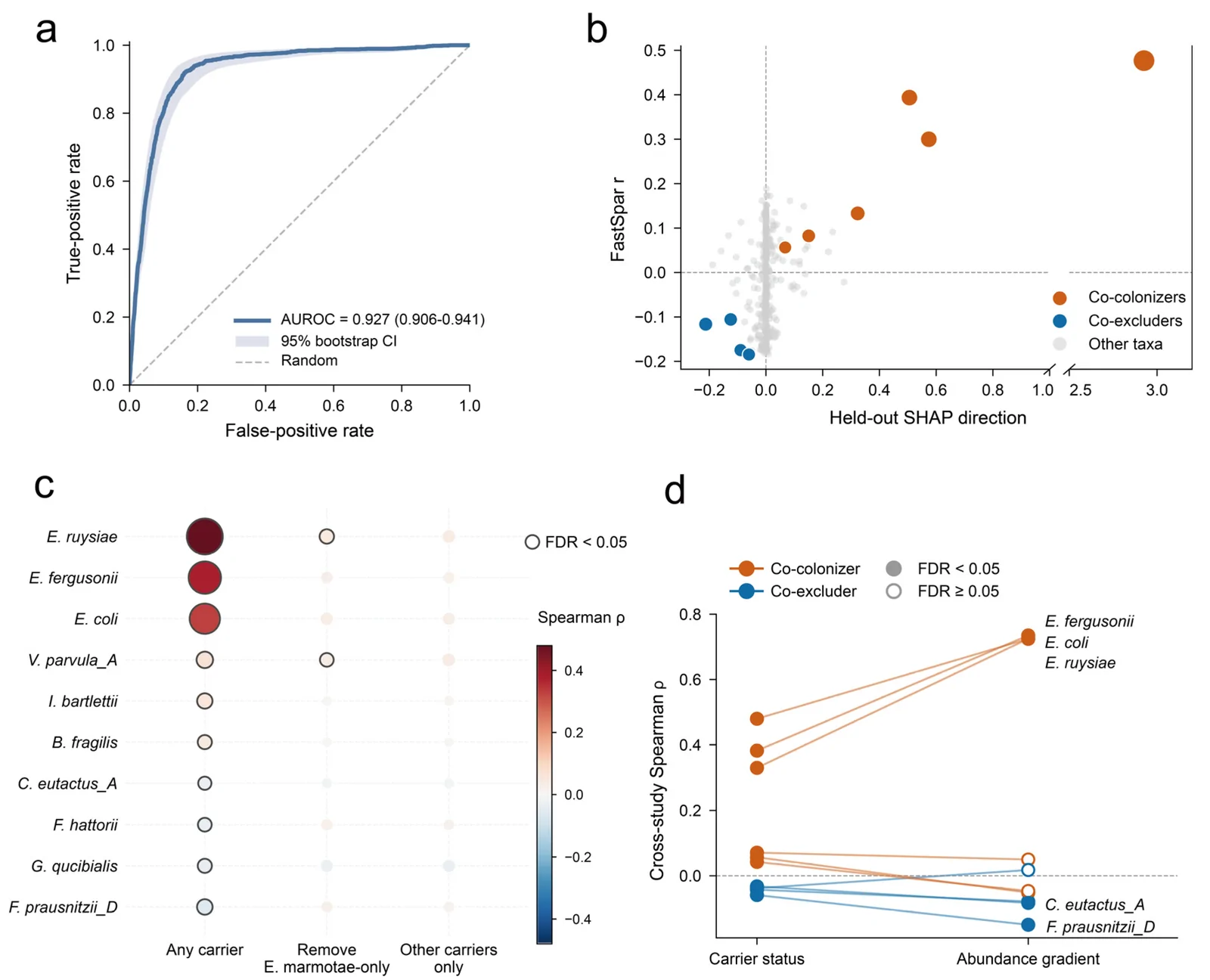
Cross-study microbial associations with *cdtABC* carriers. (**a**) Leave-one-study-out receiver operating characteristic curve for classifying carrier-detected and carrier-undetected samples from the carrier-depleted microbiome matrix. (**b**) Integration of held-out SHAP contributions and FastSpar correlations with *cdtABC* carrier abundance. Grey points represent all evaluated non-carrier taxa, whereas red and blue points indicate candidate co-colonizers and co-excluders, respectively. Point size reflects mean absolute SHAP importance. (**c**) Cross-study associations of the candidate taxa with carrier status under the any-carrier, remove E. marmotae-only and other carrier-only definitions. Circle color and size represent the direction and absolute magnitude of the pooled Spearman correlation coefficient, respectively; black outlines indicate q < 0.05. (**d**) Comparison of candidate associations with binary carrier status and the total carrier abundance gradient among carrier-detected samples. Orange and blue denote co-colonizers and co-excluders, respectively; filled and open circles indicate FDR-significant and non-significant associations. Lines provide visual guides connecting estimates for the same taxon between carrier-status and carrier-abundance models.

To test whether the presence of predicted *cdtABC* carriers could be predicted from the surrounding gut microbial community, we constructed a carrier-depleted microbiome matrix by removing the 17 carrier genomes and other genomes assigned to the same species. The resulting matrix comprised 321 non-carrier species across 12,238 samples. A leave-one-study-out classifier distinguished the 1336 carrier-detected samples from the 10,902 carrier-undetected samples with an area under the receiver operating characteristic curve (AUROC) of 0.927 (95% confidence interval, 0.906–0.941) (Figure 4a). Because the preceding analysis showed that *Escherichia marmotae* dominated the carrier-detected landscape, we next examined whether this predictive signal was confined to the *E. marmotae*-associated community background. After excluding the 1155 samples containing only the *E. marmotae* carrier, the classifier retained an AUROC of 0.806 for the remaining 181 carrier-detected samples. When the positive group was further restricted to the 159 samples containing other carriers without *E. marmotae*, the AUROC was 0.786 (Supplementary Figure S6). Although predictive performance decreased under both sensitivity definitions, the models retained above-random discrimination. These results indicated that the carrier-depleted gut microbiome contained information predictive of *cdtABC* carrier presence, with the strongest signal corresponding to the predominant *E. marmotae*-associated state but a weaker signal remaining for other carriers.

We integrated held-out SHAP contributions with FastSpar and study-stratified Spearman analyses performed across all 321 non-carrier taxa. Of the 13 taxa that passed the initial SHAP and FastSpar screening, ten were supported by both globally corrected association analyses and showed concordant directions across SHAP, FastSpar and pooled Spearman estimates (Figure 4b). Six taxa were positively associated with carrier detection, including *Escherichia ruysiae*, *E. fergusonii*, *E. coli*, *Veillonella parvula_A*, *Intestinibacter bartlettii* and *Bacteroides fragilis*. Four taxa were negatively associated, comprising *Coprococcus eutactus_A*, *Faecalibacterium hattorii*, *Gemmiger qucibialis* and *F. prausnitzii_D*. The strongest pooled associations were observed for *E. ruysiae* (Spearman’s ρ = 0.480), *E. fergusonii* (ρ = 0.382) and *E. coli* (ρ = 0.329). After samples containing only the *E. marmotae* representative were excluded, *E. ruysiae* and *V. parvula_A* remained significant, whereas none of the ten taxa remained significant when carrier-detected samples were restricted to those containing other carriers without *E. marmotae* (Figure 4c). However, the corresponding numbers of carrier-detected samples decreased from 1336 to 181 and 159, and the numbers of informative studies decreased from 56 to 34 and 33, respectively (Supplementary Figure S7a). Across the ten taxa, the median absolute pooled correlation decreased from 0.058 in the complete analysis to 0.023 and 0.017 in the two reduced analyses (Supplementary Figure S7b). Repeated downsampling produced median empirical recovery probabilities of 54.2% and 49.2% at the two reduced sample sizes (Supplementary Figure S7c). The reduced number of significant associations therefore reflects both changes in carrier composition and reduced statistical sensitivity.

Because binary carrier detection does not indicate whether associated taxa vary with carrier abundance, we next examined the ten globally robust taxa along the total carrier abundance gradient within carrier-detected samples (Figure 4d). Five taxa showed significant abundance gradient associations. The three *Escherichia* species were strongly positively associated with carrier abundance, consistent with the predominance of *E. marmotae* in the total carrier signal. By contrast, the abundances of *F. prausnitzii_D* (ρ = −0.150, q = 1.45 × 10^−6^) and *C. eutactus_A* (ρ = −0.083, q = 0.023) decreased as total carrier abundance increased. All five taxa retained the association direction observed in the carrier detection analysis. Together, the predicted catalogue, global metagenomic survey and community analyses revealed a taxonomically restricted yet geographically widespread *cdtABC* carrier landscape in the human gut that was dominated by *E. marmotae*, with carrier detection and abundance associated with reproducible variation in the surrounding microbial community.

## 4. Discussion

In this study, we combined genome screening with analyses of human gut metagenomes at the population level to determine which bacteria carry intact *cdtABC* systems and how these carriers are distributed in the gut microbiome. Previous knowledge of the taxonomic distribution of CDT has come largely from studies of individual bacterial species. Functional *cdtABC* systems have been experimentally characterized in *Campylobacter jejuni*, selected diarrhoeagenic *Escherichia coli*, *Haemophilus ducreyi*, *Aggregatibacter actinomycetemcomitans* and Helicobacter hepaticus [11,31,32,33]. These studies established the biological activity of CDT in selected isolates but did not provide a consistent basis for comparing the distribution of intact *cdtABC* loci across bacterial taxa. Our screening shows why such a basis is important. Although preliminary CDT subunit signals occurred in genomes from seven phyla and 28 orders, strict loci that met all requirements for subunit annotation, conserved domains and genomic adjacency were confined to 63 species, 14 genera and three orders within Campylobacterota and Pseudomonadota. Moreover, 726 genomes encoded a CdtB homologue supported by conserved domain evidence without carrying the complete system of three subunits. These genomes could therefore have been counted as carriers in a survey based on CdtB alone. The strict catalogue recovered established CDT producers and expanded the candidate range to less extensively studied genera, including *Edwardsiella*, *Bisgaardia*, *Frederiksenia*, *Mergibacter* and *Pasteurella*. It also revealed distinct patterns among major carrier lineages. Campylobacter contributed the largest numbers of carrier genomes and operon haplotypes, whereas *Glaesserella* showed frequent locus duplication. By contrast, Escherichia showed low prevalence across the genus but substantial sequence diversity among its carrier genomes. The 275 exact operon haplotypes further demonstrate that the catalogue captures broad sequence diversity rather than repeated examples of a few reference toxins. The phylogenetic and genomic neighbourhood analyses further strengthen the catalogue by relating operon diversity to carrier taxonomy, identifying exact haplotypes shared across species and distinguishing genomic architectures associated with particular lineages. The eight exact operon haplotypes shared across RefSeq species labels may reflect recent horizontal transfer, strong sequence conservation or taxonomic uncertainty among closely related genomes.

The Unified Human Gastrointestinal Genome resource greatly expanded the genomic representation of both cultured and uncultured gut bacteria [34]. Previous work using the global metagenomic collection analysed here described the distribution and ecological organisation of Enterobacteriaceae at both family and species levels [30]. By connecting our genome catalogue to this metagenomic collection, we detected *cdtABC* carriers across most cohorts and geographical regions, indicating that these organisms are widely distributed in the human gut. However, the observed detection rates varied among regions, and part of this variation may reflect differences in cohort composition and laboratory protocols among the contributing studies; therefore, country and continent summaries were treated as descriptive. At the species level, the carrier signal was strongly dominated by *E. marmotae*. Although *C. jejuni* is prominent in experimental studies of CDT, it was uncommon in the metagenomic collection. This contrast suggests that the species receiving the most attention in mechanistic studies are not necessarily the most prevalent *cdtABC* reservoirs in human populations. *E. marmotae* was first isolated from Himalayan marmot faeces and was later shown to possess invasive traits and other features associated with virulence [35,36]. Its subsequent isolation from healthy human faeces further confirmed its ability to colonise the human gut [37]. Our findings extend this work by showing that a *cdtABC* carrier belonging to *E. marmotae* is geographically widespread in human gut metagenomes. Moreover, the predominance of a single carrier in most carrier-detected samples suggests that *cdtABC* occurrence usually reflects read detection by one dominant lineage rather than the coexistence of multiple carriers.

The occurrence of *cdtABC* carriers was associated with reproducible differences in the surrounding gut microbial community. These associations remained evident after carrier genomes and all other genomes assigned to the same species were removed from the taxonomic profiles, indicating that they were not driven solely by the carrier species. Much of the community signal was linked to the predominant *E. marmotae* representative, although a weaker signal persisted after its exclusion. Because this exclusion substantially reduced the numbers of carrier-detected samples and informative studies, the accompanying loss of statistical significance may partly reflect an increased risk of Type II error. Positive associations with other *Escherichia* species probably reflect ecological preferences shared among closely related lineages. Conversely, the repeated negative association with *Faecalibacterium* is compatible with its reported anti-inflammatory role and with evidence that commensal communities can restrict Enterobacteriaceae through competition for nutrients and ecological niches [38,39,40]. These explanations remain untested, as the observed negative association does not establish a direct interaction between *Faecalibacterium* and *cdtABC* carriers. By integrating predictive contributions across studies with abundance correlations, we narrowed these broader community patterns to a focused set of taxa supported by complementary analyses. These taxa provide candidates for investigating the ecological interactions that influence the establishment or persistence of *cdtABC* carrier lineages.

These findings should be interpreted in light of two limitations. First, screening complete RefSeq genomes prioritized operon integrity but favored culturable isolates. Although using the UHGG catalogue partially mitigated this in the human gut, unculturable taxa in wider reservoirs remain underrepresented, making our catalogue a conservative estimate of total *cdtABC* diversity. Additionally, because short reads cannot fully resolve cryptic *Escherichia* lineages, the dominant gut signal reflects assignment to the UHGG *E. marmotae* representative cluster rather than isolate-confirmed species identity. Resolving this uncertainty will require sequencing representative human *E. marmotae* isolates, preferably using long reads, to confirm their taxonomic assignment and reconstruct the complete *cdtABC* locus structure. Second, the presence of an intact *cdtABC* locus in a genome indicates only the genetic capacity to encode the three CDT subunits and does not establish their expression, assembly into a functional toxin, secretion or genotoxic activity. Our comparisons within studies did not show a statistically significant increase in carrier detection or abundance in CRC. However, this finding does not exclude a role for CDT in colorectal tumorigenesis, as carrier detection does not directly measure toxin activity. Experimental validation in representative carrier strains will therefore be required to confirm CDT production and genotoxic activity.

Overall, our results establish a strict catalogue of intact *cdtABC* loci and reveal the distribution and microbial community context of their carriers in the human gut. Future research should prioritise representative loci from understudied carrier genera and human *E. marmotae* isolates, including functional comparisons with related *E. coli* lineages. Sequencing with long reads, combined with transcriptomic, proteomic and genotoxicity analyses, could connect carrier genomes and intact loci to active toxin production. Candidate community taxa could also be tested in coculture, organoid and gnotobiotic models to identify conditions that promote or restrict the establishment or persistence of *cdtABC* carrier lineages. Together, these findings shed light on the diversity, population distribution and ecological context of *cdtABC* carriers in the human gut. This work provides a foundation for linking predicted intact *cdtABC* loci in bacterial genomes to toxin activity and for clarifying the relevance of CDT to human health.

## Data Availability

The analysis notebooks and processed source tables used to generate the figures are publicly available on GitHub at. https://github.com/panzhuoddv/cdtabc-figures-reproducibility (accessed on 23 September 2026) The repository also includes software requirements and a data manifest for figure reproduction. All representative prokaryotic genomes analysed in this study are publicly available from the NCBI RefSeq database (https://www.ncbi.nlm.nih.gov/refseq/, accessed on 11 February 2026). Human gut reference genomes and metagenomic abundance profiles were obtained from the Unified Human Gastrointestinal Genome resource hosted by MGnify (https://ftp.ebi.ac.uk/pub/databases/metagenomics/mgnify_genomes/human-gut/, accessed on 15 March 2026). Taxonomic assignments were obtained from the Genome Taxonomy Database (https://gtdb.ecogenomic.org/, accessed on 15 March 2026). No new sequencing data were generated in this study. Processed data supporting the analyses are provided in Tables S1–S4, and source data are provided with this paper.

## Supplementary Materials

The following supporting information can be downloaded at: https://www.mdpi.com/article/10.3390/microorganisms14102244/s1, Figure S1: Stepwise identification of complete strict cdtABC loci in RefSeq genomes; Figure S2: Concordance among individual subunit and concatenated CdtABC phylogenies; Figure S3: Sampling coverage and prevalence of human gut cdtABC carriers; Figure S4: Detection and abundance of candidate cdtABC carriers across disease categories and in CRC; Figure S5: Robustness of carrier detection and *E. marmotae* predominance across abundance thresholds; Figure S6: Analyses of cross-study classification after reducing the contribution of *E. marmotae*; Figure S7: Statistical sensitivity after excluding the predominant *E. marmotae* representative; Table S1: Dataset 1; Table S2: Dataset 2; Table S3: Dataset 3; Table S4: Dataset 4.
