# Supplementary material for: Escherichia marmotae Dominates the Global Distribution of cdtABC Carriers in the Human Gut
Source: Microorganisms. 2026 Oct 3;14(10):2244. doi: 10.3390/microorganisms14102244 (PMC13707905; doi:10.3390/microorganisms14102244)
Supplement: Supplementary file 1 [file microorganisms-14-02244-s001.zip › Supplemental Materials-figures.pdf]

Supplementary Materials

Escherichia marmotae dominates the global distribution of *cdtABC* carriers in the human gut

This PDF file includes:

Supplementary Figures 1 to 7

Other Supplementary Materials for this manuscript include the following:

Supplementary Dataset 1 to 4

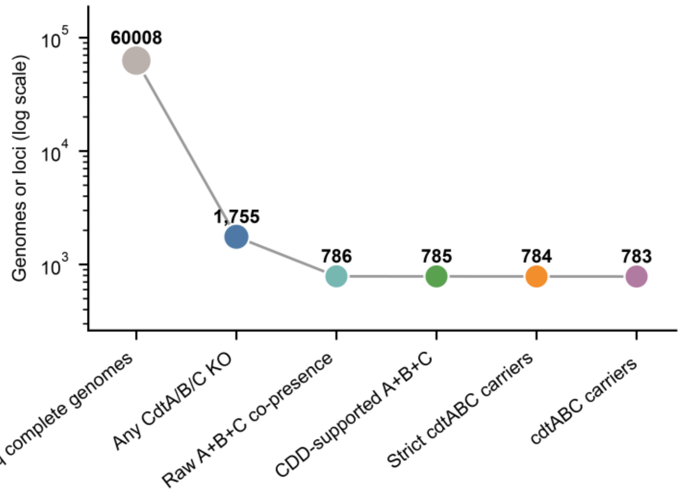

Supplementary Figure S1 Stepwise identification of complete strict *cdtABC* loci in RefSeq genomes. The workflow began with 60,008 complete RefSeq bacterial genomes. Initial KO screening identified 1,755 genomes containing at least one CdtA, CdtB or CdtC signal. Requiring all three domain-supported subunits retained 785 genomes. Requiring the three genes to occur as consecutive coding sequences on the same contig and strand identified 872 adjacent loci in 784 genomes. Exclusion of one locus containing a partial CdtB domain yielded the final catalogue of 871 complete strict *cdtABC* loci in 783 predicted carrier genomes.

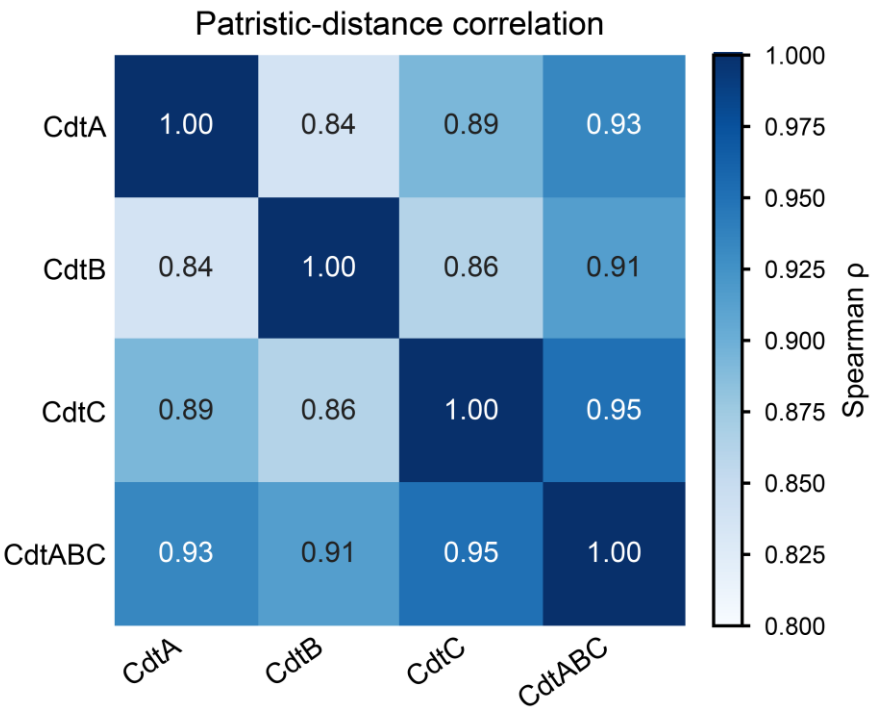

Supplementary Figure S2 Concordance among individual subunit and concatenated cdtABC phylogenies. Spearman correlations between pairwise patristic distances in the CdtA, CdtB, CdtC and concatenated CdtABC trees.

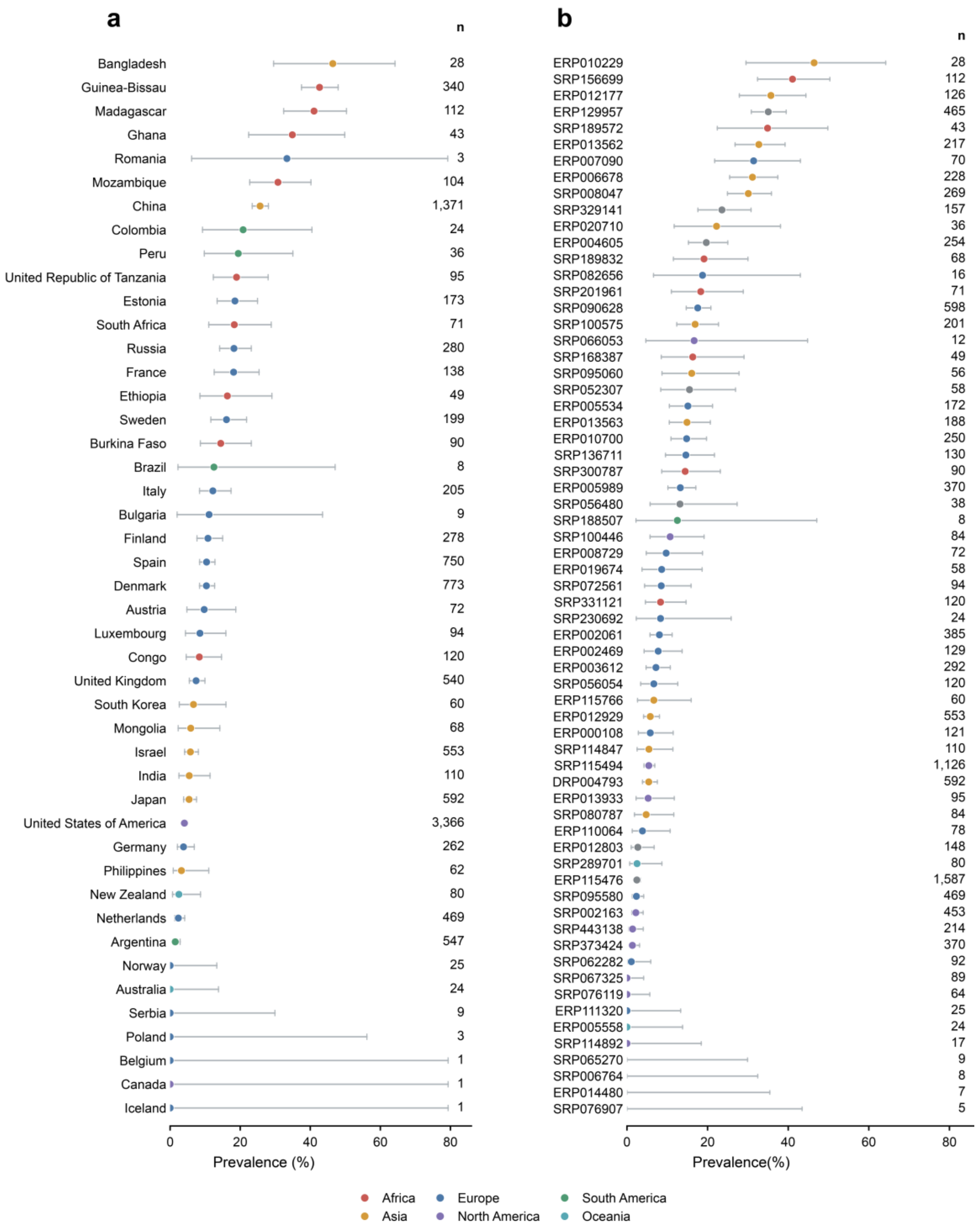

Supplementary Figure S3 Sampling coverage and prevalence of human gut cdtABC carriers. Prevalence across 45 countries (a) and 65 studies (b). Points indicate prevalence, horizontal lines indicate two sided 95% Wilson confidence intervals.

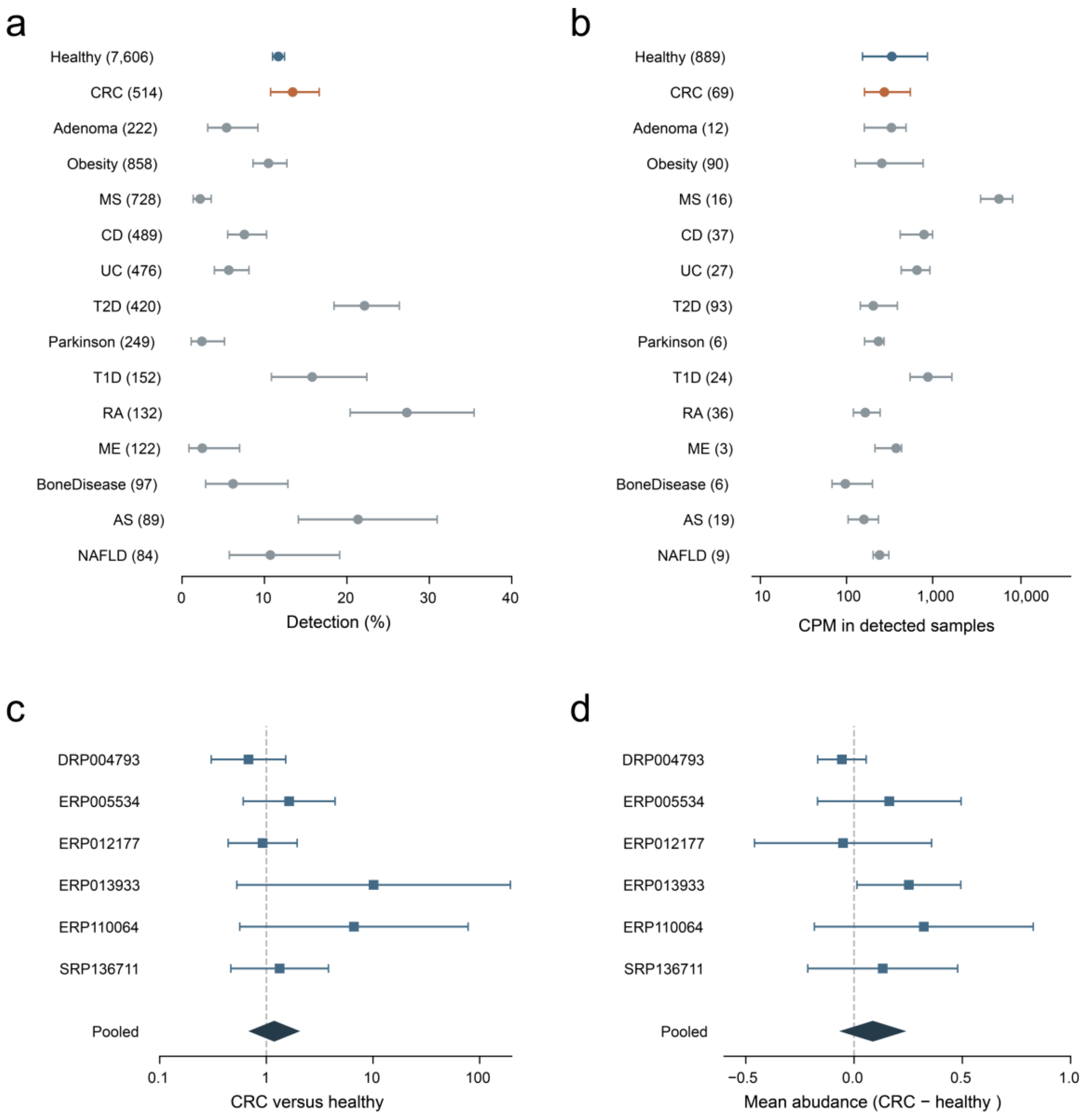

Supplementary Figure S4 Detection and abundance of candidate *cdtABC* carriers across disease categories and in CRC. a, Detection percentages across healthy samples and 14 disease categories. Points and horizontal bars indicate percentages and Wilson 95% confidence intervals, respectively; numbers in parentheses indicate total sample counts. b, Abundance among samples with detected carrier reads. Points and bars show the median and interquartile range of summed carrier CPM on a logarithmic axis; numbers in parentheses indicate detected sample counts. Healthy and CRC samples are highlighted in blue and

orange, respectively, and other categories are shown

in grey. c, CRC versus healthy detection odds ratios within six informative studies. Squares and horizontal bars indicate study estimates and 95% confidence intervals. d, Differences between CRC and healthy samples in mean $\log_{10}$(CPM + 1) within the same six studies; all samples, including those with zero carrier reads, are retained. Positive differences indicate greater transformed abundance in CRC. Diamonds in c and d show DerSimonian–Laird random effects pooled estimates and 95% confidence intervals, with two-sided nominal P values and I^2 reported below. Symbol size does not encode study weight. Dashed lines mark the null values of 1 and 0, respectively. Of eight studies containing CRC samples, one had no healthy comparator and another had no detections in either group; six studies contributed informative estimates, comprising 468 CRC and 504 healthy samples. Disease category summaries in a and b are descriptive and do not adjust for differences between contributing studies. Disease labels follow the source metadata: CRC, colorectal cancer; MS, multiple sclerosis; CD, Crohn's disease; UC, ulcerative colitis; T2D, type 2 diabetes; T1D, type 1 diabetes; RA, rheumatoid arthritis; ME, myalgic encephalomyelitis; AS, ankylosing spondylitis; NAFLD, nonalcoholic fatty liver disease. BoneDisease and Parkinson retain the source category labels. CPM, counts per million reads.

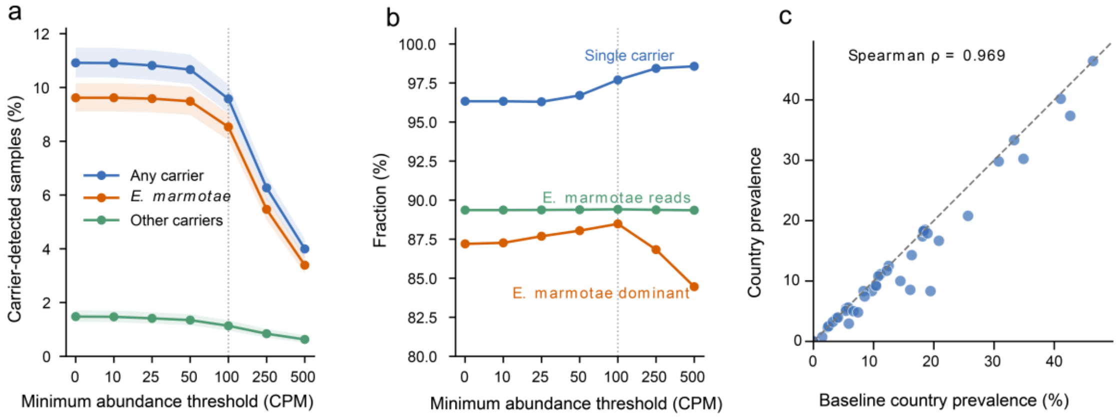

Supplementary Figure S5 Robustness of carrier detection and *E. marmotae* predominance across abundance thresholds. a, Detection prevalence of any carrier, the *E. marmotae* representative and other carriers at minimum abundance thresholds ranging from 0 to 500 CPM. Shaded regions represent Wilson 95% confidence intervals. b, Proportions of carrier detected samples containing a single carrier or dominated by the *E. marmotae* representative, together with the contribution of this representative to retained carrier reads at each threshold. c, Comparison of country prevalence estimates obtained using the baseline definition and a minimum threshold of 100 CPM. The dashed line indicates equality between the two estimates.

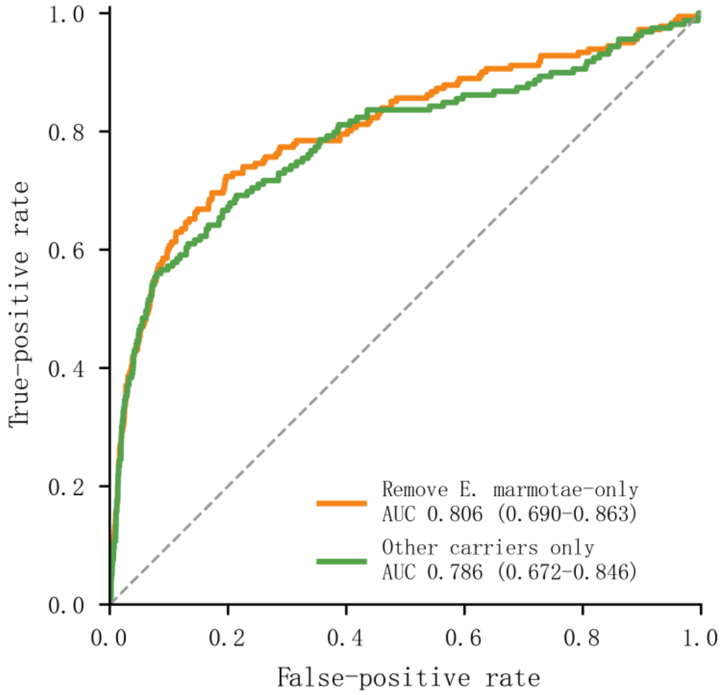

Supplementary Figure S6 Analyses of cross-study classification after reducing the contribution of *E. marmotae*.

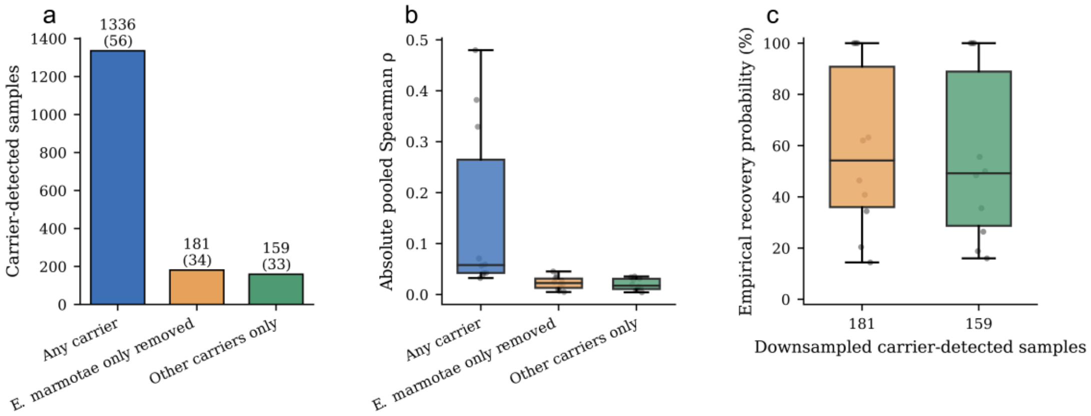

Supplementary Figure S7 Statistical sensitivity after excluding the predominant *E. marmotae* representative. a, Numbers of carrier detected samples and informative studies under the complete, remove *E. marmotae* only and other carriers only definitions. b, Pooled Spearman correlations for the ten globally robust taxa under the three carrier definitions. c, Empirical recovery probabilities for the ten globally robust taxa after repeatedly downsampling the carrier detected group to 181 or 159 samples. Recovery was defined as the proportion of 250 repetitions in which the pooled association had nominal $P < 0.05$ and the same direction as in the complete analysis.
